# Mercury Concentrations in Feathers of Albatrosses and Large Petrels at South Georgia: Contemporary Patterns and Comparisons with Past Decades

**DOI:** 10.1007/s00244-024-01067-9

**Published:** 2024-05-18

**Authors:** William F. Mills, Paco Bustamante, Francisco Ramírez, Manuela G. Forero, Richard A. Phillips

**Affiliations:** 1 British Antarctic Survey, Natural Environment Research Council, Cambridge, CB3 0ET UK; 2 Littoral Environnement et Sociétés (LIENSs), UMR 7266, CNRS-La Rochelle Université, 2 rue Olympe de Gouges, 17000 La Rochelle, France; 3 Departament de Recursos Marins Renovables, Institut de Ciències del Mar (ICM-CSIC), Passeig Marítim de la Barceloneta, 37-49, 08003 Barcelona, Spain; 4 Department of Conservation Biology, Estación Biológica de Doñana, Avda. Américo Vespucio, 26, Isla de la Cartuja, 41092 Seville, Spain; 5 Present Address: Department of Geography and Environmental Science, University of Reading, Reading, UK

## Abstract

Mercury (Hg) is an environmental contaminant that can negatively impact the health of humans and wildlife. Albatrosses and large petrels show some of the highest levels of Hg contamination among birds, with potential repercussions for reproduction and survival. Here, body feather total Hg (THg) concentrations were determined in breeding adults of five species of albatrosses and large petrels in the foraging guild at South Georgia during the mid-2010s. We tested the effects of species, sex and trophic ecology (inferred from stable isotopes) on THg concentrations and compared our results with published values from past decades. Feather THg concentrations differed significantly among species (range: 1.9–49.6 µg g^−1^ dw), and were highest in wandering albatrosses *Diomedea exulans*, intermediate in black-browed albatrosses *Thalassarche melanophris* and northern giant petrels *Macronectes halli*, and lowest in southern giant petrels *M. giganteus* and white-chinned petrels *Procellaria aequinoctialis*. Females were more contaminated than males in all species, potentially due to differences in distributions and diet composition. Across species, THg concentrations were not correlated with feather δ^13^C or δ^15^N values, implying that species effects (e.g., breeding and moulting frequencies) may be more important than trophic effects in explaining feather THg concentrations in this foraging guild. Within species, the only significant correlation was between THg and δ^13^C in wandering albatrosses, which could reflect higher Hg exposure in subtropical waters. Comparisons with THg concentrations from past studies, which reflect contamination from 10 to > 60 years ago, revealed considerable annual variation and some evidence for increases over time for wandering and black-browed albatrosses since before 1950 and from the late 1980s, respectively.

Mercury (Hg) contamination of marine ecosystems is an important environmental issue. Hg entering the environment can be of natural origin (e.g., from volcanism, rock weathering, hydrothermal vents) or anthropogenic, and human inputs have greatly increased the amount of Hg in circulation since the Industrial Revolution (Pirrone et al. 2010; Lamborg et al. 2014; Outridge et al. 2018; Streets et al. 2019). Currently, artisanal and small-scale gold mining (ASGM) is the largest anthropogenic source of Hg released into the atmosphere (Keane et al. 2023). Hg emissions are assumed to be deposited predominantly within the same hemisphere, given that the atmospheric lifetime of 3–6 months is shorter than the timescale for interhemispheric air exchange (Fisher et al. 2023; Schneider et al. 2023). Hg primarily enters the open ocean via atmospheric deposition (Driscoll et al. 2013), and once in this environment, inorganic Hg (iHg) is methylated to organic methyl-Hg (MeHg), principally by microorganisms (Hg methylators), such as iron- and sulfate-reducing bacteria (Hsu-Kim et al. 2013). MeHg, which is the most toxic and bioavailable form of Hg, bioaccumulates within marine organisms, such that concentrations increase in tissues over time, and biomagnifies up marine food chains, increasing from lower to higher trophic levels (Seco et al. 2021). In marine ecosystems, top predators with long lifespans (including large fish, seabirds and marine mammals) can therefore accumulate high Hg concentrations in their tissues (Monteiro and Furness 1995; Chételat et al. 2020).

Feathers are frequently used as a non-destructive means of monitoring Hg contamination of seabirds (Albert et al. 2019). Moult is considered to play a major role in the elimination of MeHg (Braune 1987; Renedo et al. 2021), and Hg bound in the feathers may account for > 90% of the total body burden in some seabirds, though this figure is much lower (< 10%) for albatrosses (Braune and Gaski 1987; Kim et al. 1996). Once bound to the sulfhydryl groups of keratin molecules, Hg cannot be lost from feathers (Crewther et al. 1965; Appelquist et al. 1984). The total Hg concentrations (THg) measured in feathers are almost exclusively MeHg (> 90%) and so are often used as a proxy for MeHg (Renedo et al. 2017). Depending on the seabird species, Hg in the feathers of adults may primarily reflect remobilised Hg that has accumulated in internal tissues between successive moults (i.e., capital strategy) or MeHg derived from recent dietary intake (i.e., income strategy) (Cherel et al. 2018).

Diet analyses can reveal the drivers of contamination levels, as seabirds are mainly exposed to Hg via their prey. Stomach contents or pellets can be used to identify ingested prey to species level, but are biased by the different digestion rates and retention times of prey, and cannot be collected outside of the breeding period if birds are far from the colony (Barrett et al. 2007). Stable isotope analysis offers an alternative approach for relating diet to contaminant burdens, as the isotopic composition of consumer tissues relates predictably to that of their prey (Thompson et al. 1998). Bulk carbon stable isotope values (δ^13^C) of seabird tissues vary little with trophic level (~ 1‰), but can be used to infer feeding areas (e.g., the relative dependence on inshore vs. offshore, benthic vs. pelagic diets, and on latitude/water mass), whereas those of nitrogen (δ^15^N) show a stepwise increase with trophic level (~ 3–5‰) (Peterson and Fry 1987; Hobson and Clark 1992; Bearhop et al. 2002; Cherel and Hobson 2007; Phillips et al. 2009). However, baseline δ^15^N values also vary spatially, which can obscure variation associated with trophic position (Elliott et al. 2021). For instance, high δ^13^C and δ^15^N values of feathers from adult seabirds in the southwest Atlantic Ocean indicate that they feed in neritic waters (Phillips et al. 2009; Mills et al. 2024). Stable isotope values of feathers reflect diet during their synthesis, and because they are metabolically inert, they retain this information indefinitely (Cherel et al. 2000). Albatrosses and petrels generally do not breed and moult concurrently (Prince et al. 1993; Cherel et al. 2000; Catry et al. 2013); hence, stable isotope analyses of adult feathers provide dietary information during the nonbreeding period (Cherel et al. 2000; Phillips et al. 2009).

In this study, we investigated the dynamics of Hg contamination among adults of five species of albatrosses and large petrels from the globally important populations at South Georgia, sampled in the mid-2010s. South Georgia is located ~ 300 km south of the Antarctic Polar Front (APF) in the southwest Atlantic Ocean sector of the Southern Ocean. All study species have been tracked in previous years using geolocators (Phillips et al. 2005, 2006; González-Solís et al. 2008; Clay et al. 2018; Granroth-Wilding and Phillips 2019), and we used body feather δ^13^C and δ^15^N values to infer feeding areas of individuals in this study during the non-breeding season. Our study species show some of the highest feather THg concentrations among birds; indeed, albatrosses are the most contaminated avian family in terms of Hg (Cherel et al. 2018). Our objectives were to: (i) identify different factors driving feather THg concentrations within this foraging guild (including species, sex and trophic ecology), hypothesising that species characteristics (e.g. breeding and moulting frequencies) are more important than trophic effects (Stewart et al. 1999; Anderson et al. 2009); and (ii) compare contamination levels in the 2010s (measured here), with data from past studies at South Georgia (Thompson et al. 1993; Anderson et al. 2009; Becker et al. 2002, 2016). The latter is especially pertinent as there is evidence for increasing feather THg concentrations of adult grey-headed albatrosses *Thalassarche chrysostoma* from South Georgia since the late 1980s (Mills et al. 2020a). Moreover, even at low levels, Hg contamination can have a variety of negative effects on seabirds, including on physiology, immune status and behaviour (Tartu et al. 2015; Ibañez et al. 2024), and can ultimately impact breeding success and population dynamics (Mills et al. 2020a; Goutte et al. 2014a, 2014b). Hg contamination during the non-breeding period may also have negative carry-over effects in the subsequent breeding period (Mills et al. 2020a; Carravieri et al. 2023).

## Materials and Methods

### Study Site, Species and Feather Sampling

Feather sampling of albatrosses and large petrels was undertaken at Bird Island, South Georgia (54°00’S, 38°03’W). South Georgia is a United Kingdom (UK) Overseas Territory at the northern limit of the Scotia Sea. Random samples of body feathers were obtained from breeding adults during the incubation or brood-guard periods of the following species: wandering albatross *Diomedea exulans* (*n* = 15), black-browed albatross *T. melanophris* (*n* = 15), northern giant petrel *Macronectes halli* (*n* = 16), southern giant petrel *M. giganteus* (*n* = 16) and white-chinned petrel *Procellaria aequinoctialis* (*n* = 12) (Table 1). Southern giant petrels were sampled in the 2011/2012 breeding season and other species in 2014/2015. Body feathers present less variation in Hg levels than flight feathers and their collection does not impair flight performance (Furness et al. 1986). Feathers were stored dry in sealed plastic bags or envelopes and then returned to the British Antarctic Survey (Cambridge, UK) for laboratory analyses. Birds were sexed from records of observed copulatory position, bill sizes or from DNA extracted from blood samples (Fridolfsson and Ellegren 1999). Birds were of unknown age; however, previous studies have not found significant relationships between feather THg concentrations and age in breeding adult albatrosses (Tavares et al. 2013; Bustamante et al. 2016; Mills et al. 2020a).

**Table 1:** Mean (± SD), minimum and maximum total Hg concentrations (µg g^−1^ dw) and stable isotope values (‰) of carbon (δ^13^C) and nitrogen (δ^15^N) in body feathers of albatrosses and large petrels at Bird Island, South Georgia (southwest Atlantic Ocean sector of the Southern Ocean)

		THg (µg g^−1^ dw)		δ^13^C (‰)		δ^15^N (‰)	
Species	***n***	Mean ± SD	Range	Mean ± SD	Range	Mean ± SD	Range
**Black-browed albatross**	**15**	**7.98 ± 2.87**	**2.20 to 13.20**	**− 15.8 ± 0.9**	**− 18.1 to − 14.4**	**16.2 ± 1.0**	**14.7 to 17.7**
Female	8	9.02 ± 2.94	3.40 to 13.20	− 15.8 ± 0.8	− 16.6 to − 14.4	15.9 ± 1.1	14.7 to 17.7
Male	7	6.79 ± 2.46	2.20 to 9.51	− 15.8 ± 1.2	− 18.1 to − 14.8	16.6 ± 0.8	15.5 to 17.4
**Northern giant petrel**	**16**	**8.22 ± 5.15**	**2.60 to 19.87**	**− 18.6 ± 0.7**	**− 19.8 to − 17.1**	**14.7 ± 0.8**	**12.9 to 15.9**
Female	8	9.16 ± 4.77	3.25 to 17.70	− 18.8 ± 0.6	− 19.8 to − 18.1	14.5 ± 1.0	12.9 to 15.6
Male	8	7.29 ± 5.67	2.60 to 19.87	− 18.4 ± 0.7	− 19.4 to − 17.1	14.9 ± 0.6	14.3 to 15.9
**Southern giant petrel**	**16**	**5.38 ± 1.65**	**2.62 to 8.57**	**− 21.2 ± 1.7**	**− 23.8 to − 17.7**	**13.2 ± 1.7**	**10.6 to 16.1**
Female	9	6.29 ± 1.35	4.50 to 8.57	− 20.7 ± 2.1	− 23.8 to − 17.7	13.9 ± 2.0	10.6 to 16.1
Male	7	4.21 ± 1.22	2.62 to 6.12	− 21.8 ± 0.5	− 22.6 to − 21.3	12.4 ± 0.8	11.1 to 13.2
**Wandering albatross**	**15**	**31.91 ± 11.44**	**16.38 to 49.56**	**− 18.2 ± 0.9**	**− 19.9 to − 16.6**	**15.8 ± 0.7**	**14.7 to 17.4**
Female	7	39.60 ± 7.92	27.72 to 49.56	− 17.6 ± 0.7	− 18.3 to − 16.6	15.9 ± 0.6	15.1 to 16.4
Male	8	25.17 ± 9.85	16.38 to 46.36	− 18.7 ± 0.9	− 19.9 to − 17.5	15.8 ± 0.9	14.7 to 17.4
**White-chinned petrel**	**12**	**4.87 ± 2.47**	**1.91 to 9.42**	**− 16.7 ± 0.5**	**− 18.1 to − 16.2**	**17.7 ± 1.0**	**16.1 to 19.5**
Female	8	5.33 ± 2.58	1.91 to 9.42	− 16.6 ± 0.3	− 17.0 to − 16.2	17.5 ± 1.0	16.1 to 18.4
Male	4	3.95 ± 2.28	2.05 to 7.24	− 17.0 ± 0.8	− 18.1 to − 16.4	18.0 ± 1.2	16.8 to 19.5

Feather samples were collected from all species in the 2014/2015 breeding season, except southern giant petrels *Macronectes giganteus*, which were sampled in 2011/2012

All study species (excluding the wandering albatross) typically breed annually and during the austral summer, returning to the colony from September to November and fledging chicks from March to June. Wandering albatrosses, however, return from October to November, fledge chicks from November to December in the following year, and breed biennially if successful. Moult and breeding are energetically expensive and do not tend to occur simultaneously in albatrosses and petrels (Prince et al. 1993; Cherel et al. 2000; Catry et al. 2013). However, some body feather moult occurs during the early breeding season in giant petrels, and to a limited extent during the late breeding season in black-browed albatrosses at South Georgia (Hunter 1984; Catry et al. 2013). Growing body feathers, which were present in a few giant petrels, were avoided during sampling. Two generations of body feathers were always apparent, but only the newer, less abraded feathers were collected, hence it is likely that these feathers represent the preceding non-breeding period in all cases. Body feather replacement in the study species occurs gradually during the non-breeding period (~ 7% being moulted and replaced at any one time), and so the exact timing of moult of individual feathers is unknown (Battam et al. 2010).

### Total Mercury Analysis

Feathers were cleaned using repeated chloroform:methanol solution (2:1 v/v) and Milli-Q® water rinses. The feather samples were then air-dried under a fume hood for 48 h and cut into very fine fragments using stainless steel scissors. Multiple feathers were pooled and homogenised per individual to ensure compatibility with previous studies at South Georgia (Thompson et al. 1993; Anderson et al. 2009; Becker et al. 2002, 2016). THg concentrations were measured in subsamples of the homogenised body feathers using an Advanced Mercury Analyser spectrophotometer (AMA 254 Altec®) at the laboratory Littoral Environnement et Sociétés (LIENSs, La Rochelle, France). Each sample was analysed in duplicate or triplicate until the relative standard deviation (RSD) between measurements was < 10%. Blanks were analysed at the beginning of each sample run and accuracy was assessed using a certified reference material (CRM), lobster hepatopancreas TORT-3 (National Research Centre [NRC], Canada; certified THg concentration: 0.292 ± 0.022 µg g^−1^ dw), and our measured concentration was 0.295 ± 0.006 µg g^−1^ dw (*n* = 27). Our CRM results were in thus in good agreement with the certified values with a recovery of 101.1 ± 2.1%. The detection limit of the AMA was 0.005 µg g^−1^ dw. THg concentrations are presented in µg g^−1^ dw.

### Stable Isotope Analysis

Stable isotopes of carbon and nitrogen were measured in the same homogenised feather subsamples as above. Subsamples were weighed (~ 0.3 mg) into 6 × 4 mm tin capsules using a microbalance and stable isotope analyses were undertaken at the Laboratory of Stable Isotopes at the Doñana Biological Station (Seville, Spain). Samples were combusted at 1020 °C with a continuous flow isotope-ratio mass spectrometry system by means of Flash HT Plus elemental analyser coupled to a Delta-V Advantage isotope ratio mass spectrometer via a CONFLO IV interface (ThermoFisher Scientific, Bremen, Germany). Stable isotope ratios are reported using the conventional δ notation (‰) following the equation: δX = [(R_sample_/R_standard_) − 1] × 1000, where X is ^13^C or ^15^N, R is the corresponding ratio ^13^C:^12^C or ^15^N:^14^N, and R_standard_ is the ratio of international references Vienna Peedee Belemnite for carbon and atmospheric N_2_ (AIR) for nitrogen. The following internal standards were used: EBD-23 (cow horn), LIE-BB (whale baleen), and LIE-PA (razorbill feathers). Internal standards were routinely inserted into the sampling sequence to correct for linearity and instrument drift. Replicate assays of internal standards indicated analytical precisions of ± 0.1 and ± 0.2‰ for δ^13^C and δ^15^N, respectively. Internal standards were calibrated with international standards from the International Atomic Energy Agency (IAEA, Vienna).

### Data Analysis

Data were analysed using R version 4.0.3. and visualised using the ggplot2 package (Wickham 2016; R Core Team 2020). Assumptions of normality of residuals and homogeneity of variances were tested using Shapiro–Wilk and Levene’s tests, respectively. THg concentrations were subsequently log-transformed. The effects of species, sex and their two-way interaction on feather THg concentrations were tested using a two-way ANOVA followed by post-hoc Tukey’s HSD tests. The effect of species on feather δ^13^C and δ^15^N values was testing using Kruskal–Wallis tests and post-hoc Dunn’s tests. Welch’s *t*-tests were used to assess sex differences in feather δ^13^C and δ^15^N values for each species. Spearman’s rank correlations were used to test for associations between feather THg concentrations and δ^13^C and δ^15^N values across species and for each species separately, as pooling species that migrate to distinct habitats with different isotopic baselines (e.g., oceanic vs. continental shelf/shelf-slope waters) may obscure relationships with THg (Anderson et al. 2009; Blévin et al. 2013). Lastly, we compared our data with previously published feather THg concentrations for the study species at South Georgia (Thompson et al. 1993; Anderson et al. 2009; Becker et al. 2002, 2016). Only means, SDs and sample sizes were available from these previous studies (Table 2). Statistical significance was assumed at α = 0.05 in all cases.

**Table 2:** Mean (± SDs) total Hg concentrations (µg g^−1^ dw) and stable isotope values (‰) of carbon (δ^13^C) and nitrogen (δ^15^N) in body feathers of albatrosses and large petrels sampled in different years at Bird Island, South Georgia (southwest Atlantic Ocean sector of the Southern Ocean)

Species	Year	*n*	THg (µg g^−1^ dw)	δ^13^C (‰)	δ^15^N (‰)	Citation
Black-browed albatross	1989	20	4.57 ± 1.98	–	–	Thompson et al. (1993)
	1998	16	5.39 ± 2.05	–	–	Becker et al. (2002)
	2002	16	8.35 ± 2.63	− 14.9 ± 0.9	15.9 ± 1.0	Anderson et al. (2009)
	2006	10	6.86 ± 2.87	− 15.3 ± 1.7	15.4 ± 1.8	Cherel et al. (2018)
	2015	15	7.98 ± 2.87	− 15.8 ± 0.9	16.2 ± 1.0	This study
Northern giant petrel	1998	37	4.99 ± 3.76	−	–	Becker et al. (2002)
	2002	15	10.52 ± 5.54	− 18.8 ± 0.9	13.8 ± 1.1	Anderson et al. (2009)
	2015	16	8.22 ± 5.15	− 18.6 ± 0.7	14.7 ± 0.8	This study
Southern giant petrel	1998	29	7.77 ± 3.57	–	–	Becker et al. (2002)
	2002	16	8.25 ± 3.98	− 21.0 ± 1.4	12.9 ± 1.6	Anderson et al. (2009)
	2012	16	5.38 ± 1.65	− 21.2 ± 1.7	13.2 ± 1.7	This study
Wandering albatross	Pre-1950	7	20.69 ± 15.23	–	–	Thompson et al. (1993)
	1985/89	66	19.59 ± 10.12	–	–	Thompson et al. (1993)
	2002	14	27.43 ± 8.14	− 17.3 ± 0.8	15.2 ± 0.8	Anderson et al. (2009)
	2006	10	20.31 ± 5.88	− 17.4 ± 1.1	15.4 ± 0.6	Cherel et al. (2018)
	2009	34	20.14 ± 7.64	–	–	Tavares et al. (2013)
	2015	15	31.91 ± 11.44	− 18.2 ± 0.9	15.8 ± 0.7	This study
White-chinned petrel	1998	10	3.79 ± 1.72	−	−	Becker et al. (2002)
	2002	16	7.43 ± 1.97	− 15.5 ± 0.8	17.6 ± 1.4	Anderson et al. (2009)
	2015	12	4.87 ± 2.47	− 16.7 ± 0.5	17.7 ± 1.0	This study

Samples analysed by Cherel et al. (2018) were of a single feather per bird, and Thompson et al. (1993) did not specify the exact year for feathers collected before 1950 and pooled THg concentrations of wandering albatrosses from 1985 and 1989 as they were not significantly different

## Results

### Total Hg Concentrations

**Fig. 1:**
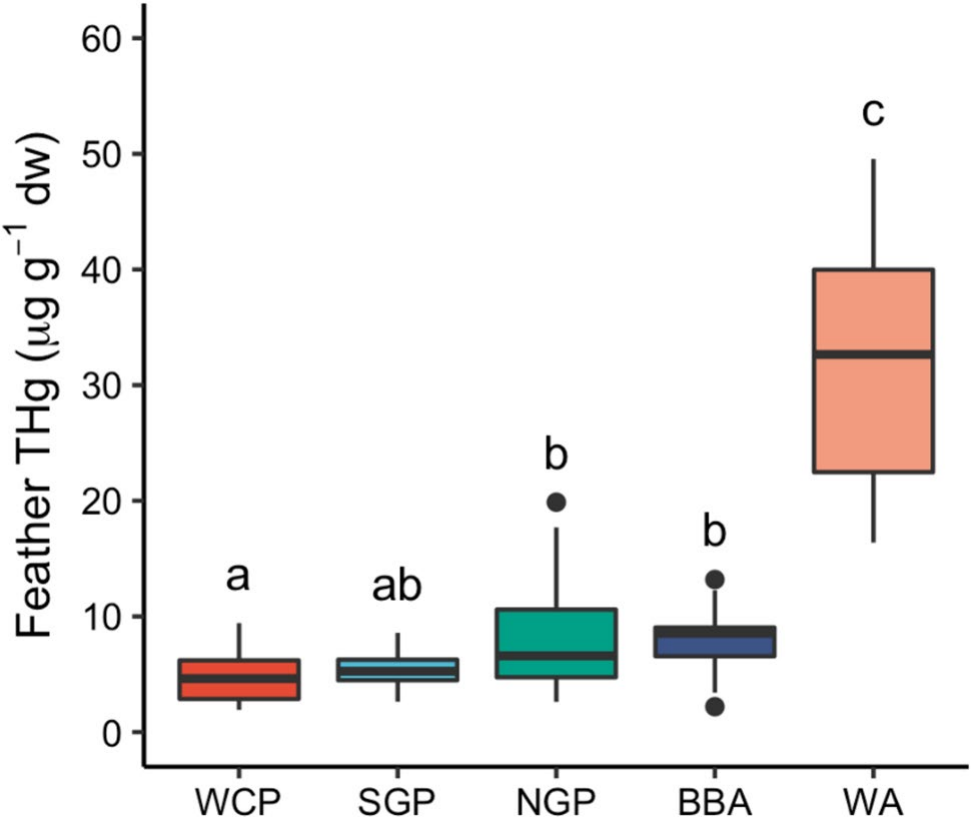
Boxplots of total Hg concentrations (µg g^−1^ dw) in body feathers of albatrosses and petrels sampled at Bird Island, South Georgia. Species abbreviations are as follows: BBA = black-browed albatross *Thalassarche melanophris*; NGP = northern giant petrel *Macronectes halli*; SGP = southern giant petrel *M. giganteus*; WA = wandering albatross *Diomedea exulans*; WCP = white-chinned petrel *Procellaria aequinoctialis*. Samples were collected from southern giant petrels in the 2011/2012 breeding season and from all other species in 2014/2015. Species sharing superscript letters are not significantly different according to post-hoc Tukey’s HSD tests. Boxplots show medians (horizontal lines), interquartile range (IQR; boxes), the lowest and highest values within 1.5 × IQR (whiskers) and outliers (black points)

There were significant effects of species and sex on (log-transformed) feather THg concentrations (two-way ANOVA, F_4,64_ = 43.1, *p* < 0.001 and F_1,64_ = 12.4, *p* < 0.001, respectively). The interaction term was not significant (F_4,64_ = 0.13, *p* = 0.97). The general pattern from the post-hoc Tukey’s HSD tests was that feather THg concentrations were considerably higher in wandering albatrosses than other species, intermediate in black-browed albatrosses and northern giant petrels, and lowest in southern giant petrels and white-chinned petrels (Fig. 1). Females had higher THg concentrations than males in all species (Table 1). In a comparison with previous studies, feather THg concentrations of all study species showed annual variation, and there was some evidence for increases over time for wandering and black-browed albatrosses since before 1950 and from the late 1980s, respectively (Table 2).

### Stable Isotopes

**Fig. 2:**
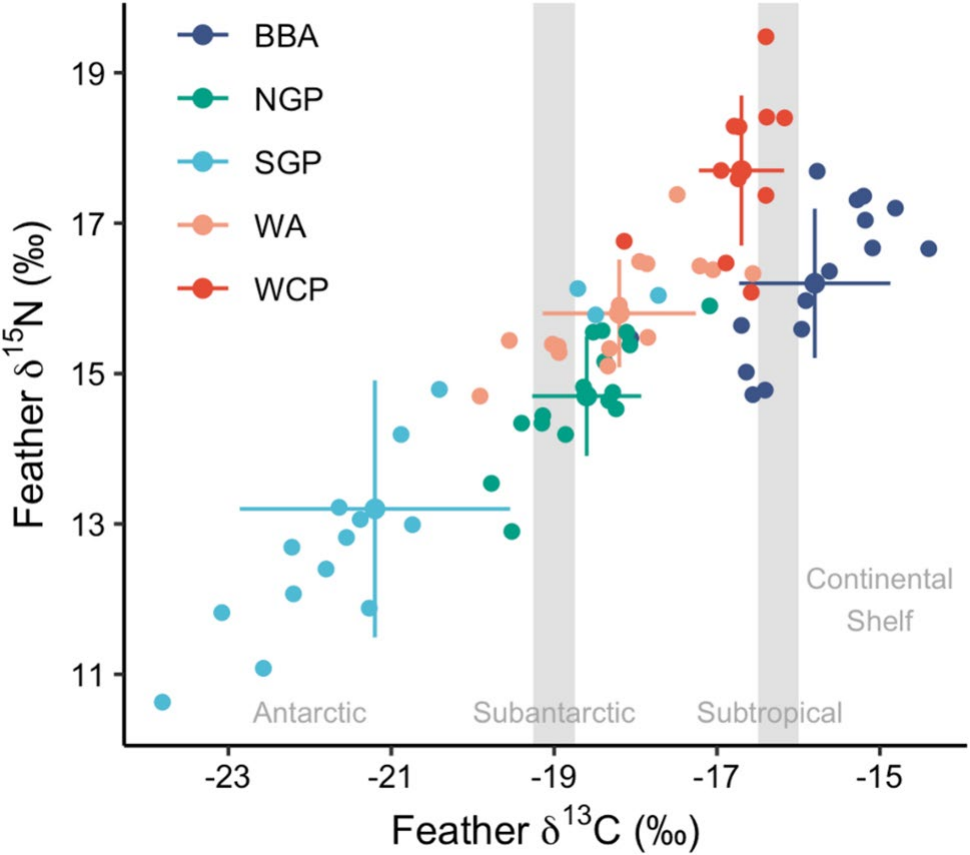
Mean (± SDs) and individual carbon (δ^13^C) and nitrogen (δ.^15^N) stable isotope values (‰) of body feathers of albatrosses and petrels sampled at Bird Island, South Georgia. Species abbreviations are: BBA = black-browed albatross *Thalassarche melanophris*; NGP = northern giant petrel *Macronectes halli*; SGP = southern giant petrel *M. giganteus*; WA = wandering albatross *Diomedea exulans*; WCP = white-chinned petrel *Procellaria aequinoctialis*. Samples were collected from southern giant petrels in the 2011/2012 breeding season and from all other species in 2014/2015. Grey vertical shaded areas and text reflect the approximate locations of biogeographic boundary zones (Phillips et al. 2009)

**Fig. 3:**
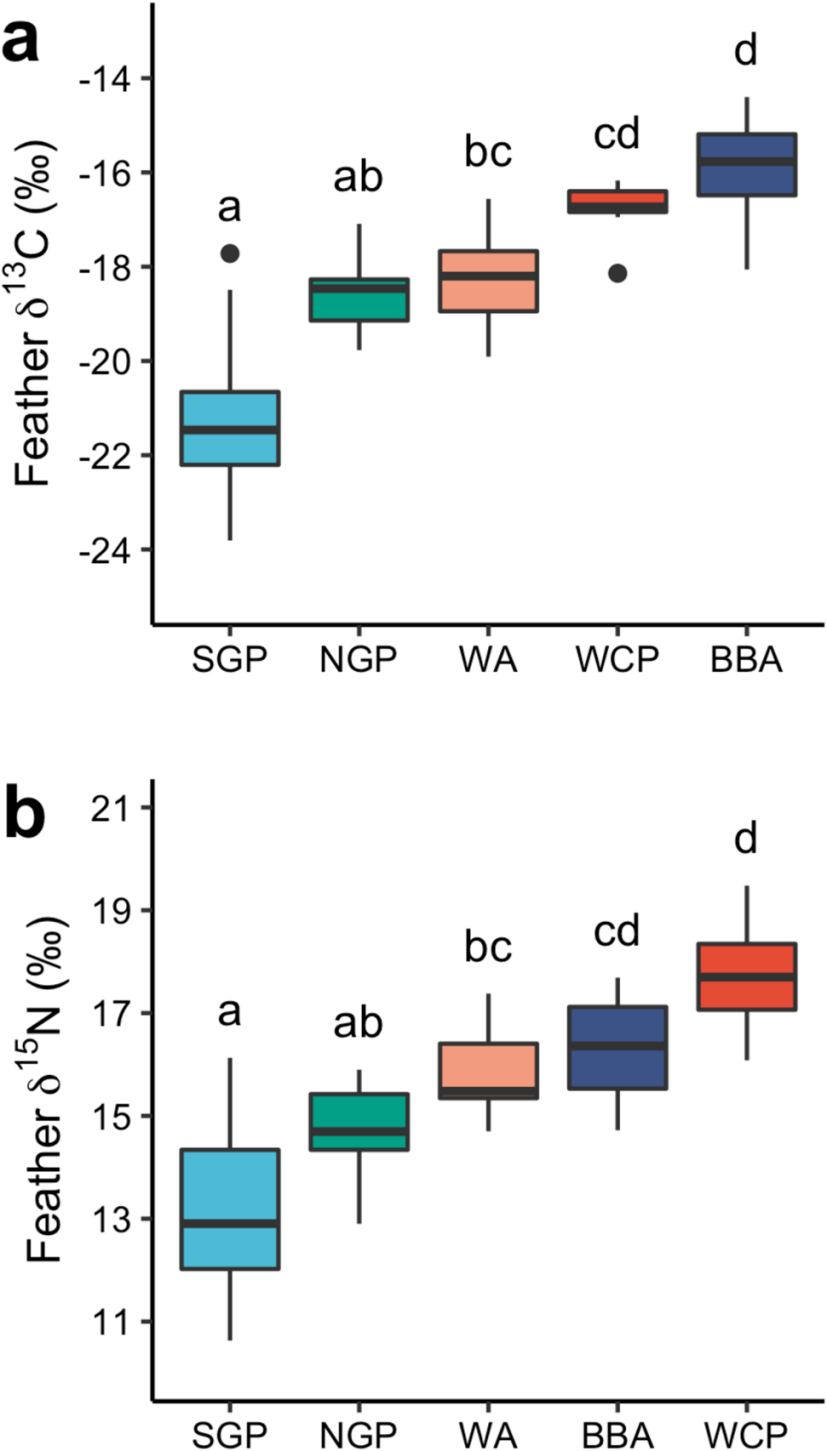
Boxplots of (**a**) carbon (δ^13^C) and (**b**) nitrogen (δ^15^N) stable isotope values (‰) in body feathers of albatrosses and large petrels sampled at Bird Island, South Georgia. Species abbreviations are: BBA = black-browed albatross *Thalassarche melanophris*; NGP = northern giant petrel *Macronectes halli*; SGP = southern giant petrel *M. giganteus*; WA = wandering albatross *Diomedea exulans*; WCP = white-chinned petrel *Procellaria aequinoctialis*. Samples were collected from southern giant petrels in the 2011/2012 breeding season and from all other species in 2014/2015. Species sharing superscript letters are not significantly different according to post-hoc Dunn’s tests. Boxplots show medians (horizontal lines), interquartile range (IQR; boxes), the lowest and highest values within 1.5 × IQR (whiskers) and outliers (black points)

**Fig. 4:**
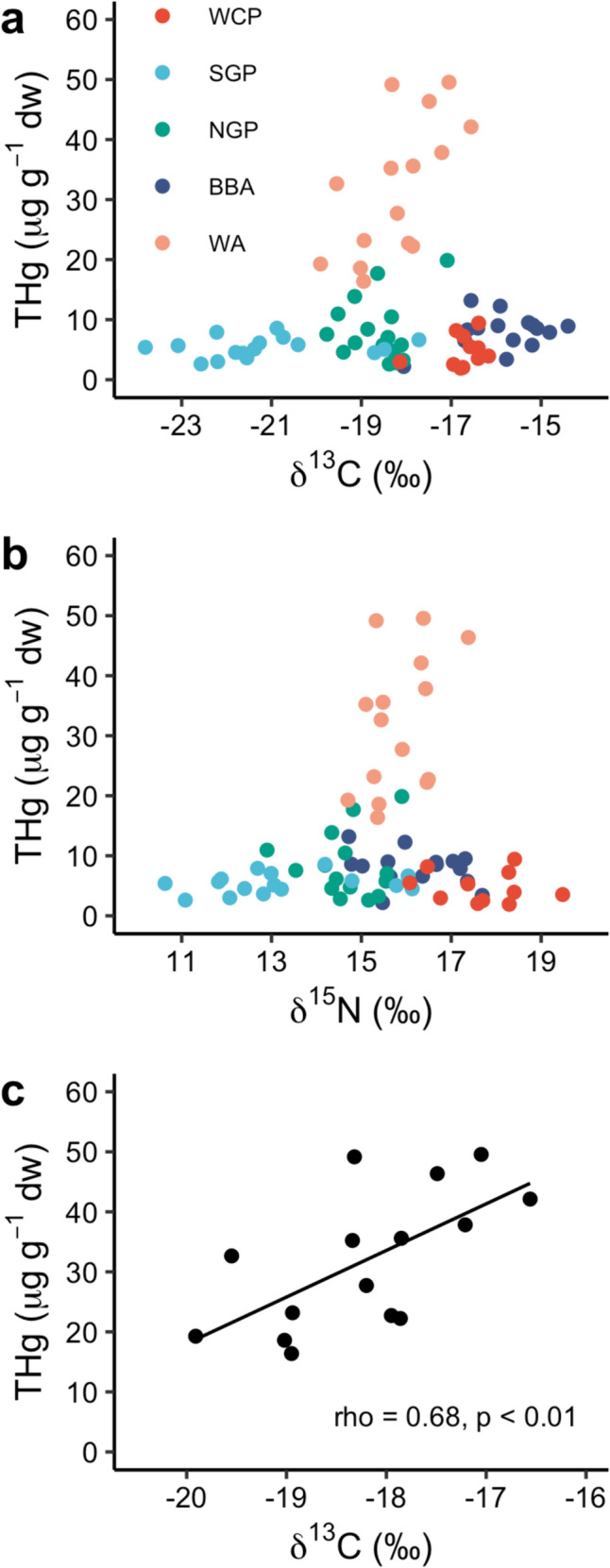
Relationships between body feather total Hg (THg) concentrations (µg g^−1^ dw) and (**a**) δ^13^C and (**b**) δ^15^N values (‰) of body feathers of albatrosses and large petrels; and (**c**) relationships between feather THg concentrations and δ^13^C values of wandering albatross *Diomedea exulans* sampled at Bird Island, South Georgia*.* Species abbreviations are: BBA = black-browed albatross *Thalassarche melanophris*; NGP = northern giant petrel *Macronectes halli*; SGP = southern giant petrel *M. giganteus*; WA = wandering albatross; WCP = white-chinned petrel *Procellaria aequinoctialis*

Species had a significant effect on feather δ^13^C and δ^15^N values (Kruskal–Wallis tests, χ^2^_4_ = 55.9, *p* < 0.0001 and χ^2^_4_ = 45.5, *p* < 0.0001, respectively) (Figs. 2, 3). Post-hoc Dunn’s tests showed that the ranking of species in order of increasing (i.e., less negative) δ^13^C values was southern giant petrel, northern giant petrel, wandering albatross, white-chinned petrel and black-browed albatross (Fig. 3), and of increasing δ^15^N values was southern giant petrel, northern giant petrel, wandering albatross, black-browed albatross and white-chinned petrel (Fig. 3). Feather δ^13^C values of female wandering albatrosses were significantly less negative than those of males (Welch’s *t*-test, *t* = 2.63, *p* < 0.05), but there were no other significant differences in feather δ^13^C and δ^15^N values for any study species (all *p* ≥ 0.15) (Table 1). Feather THg concentrations were not significantly correlated with δ^13^C or δ^15^N values across species (Spearman’s rank correlations, rho = 0.12, *p* = 0.30 and rho = 0.05, *p* = 0.68) (Fig. 4). The only significant correlation within species was between THg and δ^13^C values in wandering albatrosses (rho = 0.68, *p* < 0.01) (Fig. 4).

## Discussion

Certain life-history and ecological traits of albatrosses and large petrels (e.g., high trophic positions) lead them to accumulate high Hg concentrations in their tissues. In this study, we measured body feather THg concentrations of five species in the foraging guild of albatrosses and large petrels at South Georgia during the mid-2010s, where there are globally important breeding populations of all study species. Our study analysed the underlying drivers of variation in Hg contamination and compared the THg concentrations with those of previous studies, reflecting contamination during the previous two to over six decades.

### Interspecific Differences in Hg Contamination

Species had a significant effect on feather THg concentrations in our study. Most notably, feather THg concentrations of wandering albatrosses were far higher than our other study species (Fig. 1). THg concentrations were also higher than those of wandering albatrosses at the Crozet (mean ± SD, 22.14 ± 10.30 µg g^−1^ dw), Kerguelen (16.59 ± 3.78 µg g^−1^ dw) and Prince Edward Islands (24.83 ± 12.35 µg g^−1^ dw) (Thompson et al. 1993; Carravieri et al. 2014a; Bustamante et al. 2016; Cherel et al. 2018), and of grey-headed albatrosses sampled at Bird Island in 2013/14 (13.08 ± 6.56 µg g^−1^ dw) (Mills et al. 2020a). Among albatrosses and petrels, only the Amsterdam albatross *D. amsterdamensis* has higher mean feather THg concentrations (34.60 ± 12.50 µg g^−1^ dw) (Cherel et al. 2018).

Different moulting patterns provide one explanation for the significant interspecific differences in THg concentrations in our study, as feather THg concentrations do not just reflect exposure during synthesis (i.e., dietary intake), but also the release of Hg accumulated since the previous moult (Anderson et al. 2009; Carravieri et al. 2014b; Cherel et al. 2018). Species that take several years to moult all their feathers will accumulate Hg over a considerably longer period than those which replace all feathers annually (Stewart et al. 1999; Anderson et al. 2009). Although this seems likely to partly explains the higher Hg concentrations of wandering albatrosses compared to our other study species, which is the only biennial breeder in our study, breeding frequency (i.e., annual vs. biennial) was not an explanatory factor in models of feather THg concentrations of various albatross species in the review by Cherel et al. (2018), and feather concentrations were considered to predominantly reflect dietary intake.

An alternative explanation for the significant interspecific differences in THg concentrations is variation in diets and feeding areas, and hence dietary exposure to MeHg. However, the interspecific pattern of feather δ^13^C values did not correspond exactly with THg concentrations (Figs. 1 and 2; see below), which is likely because the different species migrate to habitats with different isotopic baselines in the Southern Ocean (Anderson et al. 2009). There is a general pattern of increasing Hg contamination of seabirds feeding in subtropical compared to subantarctic and Antarctic waters in the Southern Ocean (Renedo et al. 2020), and from species that feed in coastal to more oceanic waters (Ochoa-Acuna et al. 2002). Wandering albatrosses forage in various habitats during the non-breeding period, though mostly in subantarctic to subtropical waters within the southwest Atlantic Ocean (Fig. 2) (Phillips et al. 2009; Clay et al. 2018). Hence, their use of lower latitude foraging areas likely contributes to their high Hg contamination levels. In contrast, as reflected in our isotope data, most southern giant petrels, which showed much lower THg concentrations, remain in waters south of the APF during the non-breeding season (Fig. 2). This corresponds with previous tracking studies, which show that southern giant petrels exploit Antarctic waters to a greater extent than northern giant petrels, many of which utilise the Patagonian Shelf or subantarctic waters to the north of South Georgia (Phillips et al. 2009; González-Solís et al. 2008; Granroth-Wilding and Phillips 2019). The high feather δ^13^C and δ^15^N values of white-chinned petrels and black-browed albatrosses are indicative of feeding in continental shelf and shelf-slope regions (Fig. 2) (Phillips et al. 2009; Mills et al. 2024), with the former feeding on the Patagonian Shelf and Humboldt Upwelling System off Chile during the non-breeding period (Phillips et al. 2006), and the latter migrating to the Benguela Upwelling System off southwest Africa (Phillips et al. 2005). Many of the birds that migrate to continental shelf and shelf-slope waters will follow vessels, feeding on discarded demersal fishes which potentially have a high Hg content, possibly because they have longer lifespans and feed at higher trophic levels on prey that usually have higher Hg levels than those in the epipelagic zone (Arcos et al. 2002; Petersen et al. 2008). Our stable isotope data indicate that these descriptions of non-breeding distributions are appropriate for the individuals in our study (Fig. 2). Diet composition, including the consumption of prey from different depths in the water column or of different sizes, may also influence Hg exposure. However, conventional diet data are not available for our study species during the non-breeding period, when they are far from land and are not accessible for sampling.

### Sex Differences in Hg Contamination

There was a significant effect of sex on feather THg concentrations in our study, with females having higher levels of Hg contamination than males even though some Hg in females may be deposited in the egg (Robinson et al. 2012; Carravieri et al. 2014a). The sex-species interaction was not significant. Assuming a strong influence of dietary intake on feather THg of albatrosses and giant petrels (Cherel et al. 2018; Renedo et al. 2021), differences in contamination may be due to sex-specific differences in diets and feeding areas (Carravieri et al. 2014a; Bustamante et al. 2016). Indeed, higher feather and blood THg concentrations of female compared to male wandering albatrosses at the Crozet Islands were attributed to greater time spent foraging in subtropical and subantarctic waters than in Antarctic waters (Carravieri et al. 2014a; Bustamante et al. 2016). Our study found that feather δ^13^C values were more negative in male than female wandering albatrosses, potentially indicating higher-latitude feeding areas. A previous study at South Georgia found a weak overall effect of sex on δ^13^C in the sexually size-dimorphic albatrosses and giant petrels, although none of the comparisons within species were statistically significant (Phillips et al. 2009). Tracking studies in the Indian Ocean indicate there is some sexual segregation by latitude in core foraging areas of non-breeding wandering albatrosses, with females tending to feed in more northerly waters (Weimerskirch et al. 2014). Geolocator data from non-breeding black-browed albatrosses show that females feed 4 to 5° further north within the Benguela Upwelling system than males (Phillips et al. 2005); however, there is a degree of overlap which may be why there was no difference between sexes in mean feather δ^13^C values. There is limited evidence for sexual segregation in foraging areas of northern giant petrels during winter, although males have a larger foraging range than females; however, female southern giant petrels forage more on the southern Patagonian shelf-break and males are mostly restricted to South Georgia and more southerly waters (González-Solís et al. 2008). Hence it appears that the sex differences in feather Hg concentrations in some study species may result from differences in diet composition or other aspects of foraging behaviour or distribution that are too subtle to be reflected in the stable isotope data. Small sample sizes of some species or sex groups may have also contributed to the lack of significant differences.

### Influence of Diets and Distributions

THg concentrations were not correlated with feather δ^13^C or δ^15^N values across species (see above). The wandering albatross was the only species for which we found a significant correlation between THg concentrations and stable isotope values (Fig. 4). The relatively weak correlation with δ^13^C values may be because we analysed pooled rather than individual feathers, to ensure comparability with previous data (Cherel et al. 2018). Additionally, integration periods for stable isotopes and THg into feathers will differ if MeHg is maintained in a body reservoir until it can be eliminated (Bond 2010). However, THg in body feathers of albatrosses appears to be a faithful reflection of Hg exposure on moulting grounds (Cherel et al. 2018). In the Southern Ocean, feather δ^13^C values of wandering albatrosses should principally reflect foraging latitude, with values increasing from waters in Antarctica towards the subantarctic and subtropics (Cherel and Hobson 2007; Phillips et al. 2009). Although two studies found that MeHg concentrations were higher in waters to the south than north of the APF (Cossa et al. 2011; Yue et al. 2023), there is a well-documented pattern of increasing Hg contamination of seabirds feeding in Antarctic and subantarctic waters compared to those in the subtropics (Carravieri et al. 2016, 2017; Renedo et al. 2020; Mills et al. 2022) This may reflect spatial differences in the bioavailability of Hg to seabirds, potentially due to greater vertical mixing and more efficient Hg methylation at depth in lower latitudes (Renedo et al. 2020). Differences in food chain lengths with latitude could also contribute to these spatial differences, which are potentially shorter at higher than at lower latitudes (Forero et al. 2005; Renedo et al. 2020). There were no significant correlations between THg concentrations and δ^15^N values for any study species. Typically, δ^15^N is used as a proxy for trophic level, and therefore values are expected to correlate with THg because of biomagnification through the food web. However, moulting habitats of our study species are likely marked by variable δ^15^N baselines (St John Glew et al. 2021). Hence δ^15^N values across the study species may not relate directly to trophic level. Future studies at South Georgia could use compound-specific stable isotope analyses of amino acids to provide unbiased estimates of trophic positions (Elliott et al. 2021), as has been conducted on some seabird species elsewhere in the Southern Ocean (Thébault et al. 2021; Quillfeldt et al. 2023).

### Long-Term Changes in Hg Contamination

Data on feather THg concentrations were available from the late 1990s and early 2000s for giant petrels and white-chinned petrels, the late 1980s and the mid 2000s for black-browed albatrosses, and before 1950, the late 1980s and mid 2000s for wandering albatrosses (Table 2). However, caution should be applied when comparing data among previous studies as the methods used to quantify THg concentrations differ. For instance, one study extracted organic Hg to overcome the application of iHg as a preservative on museum specimens (Thompson et al. 1993). Distinguishing typical annual variation from genuine long-term trends is challenging because of the limited availability of previous data; nevertheless, there was some evidence for a slight increase over time in feather THg concentrations of black-browed albatrosses and wandering albatrosses since the late 1980s and before 1950, respectively (Table 2). However, annual variation was high, and trends were less convincing than for increasing Hg contamination of grey-headed albatrosses at South Georgia since the late 1980s (Mills et al. 2020a). Increasing Hg contamination could indicate increased environmental exposure within feeding areas (i.e., changes in bioavailability of MeHg within foraging areas), potentially due to increasing anthropogenic Hg emissions in the Southern Hemisphere across our study period (Streets et al. 2017). Shifts in diets and foraging areas towards more contaminated prey or regions could also explain temporal variation. Diet composition of albatrosses and petrels can be highly variable among years, at least during the breeding season (Mills et al. 2020b, 2021). Quantifying diets and foraging areas alongside levels of Hg contamination can help interpret trends, but stable isotope data were not available in all previous studies (Table 2). More work is required to understand long-term drivers and trends in contamination of seabirds in general, including the relative importance of natural and anthropogenic changes in the environment. Museum specimens may be a useful source of material for extending time series for some species and sites.

## Data Availability

The data supporting this study will be made available from the corresponding author upon reasonable request.
